# 653 The Power of Words: Leveraging Messaging to Prevent Injuries and Save Lives

**DOI:** 10.1093/jbcr/iraf019.282

**Published:** 2025-04-01

**Authors:** Rebecca Coffey, Karla Klas, Daniel Chacon

**Affiliations:** Parkland Burn Center; University of Michigan, Michigan Medicine Trauma Burn Center; Alisa Ann Ruch Burn Foundation

## Abstract

**Introduction:**

Organizations frequently disseminate safety messaging to convey crucial injury prevention information. However, a systematic evaluation of these messages for accuracy, relevance, clarity, accessibility, and efficacy is often lacking or conducted with insufficient rigor. To address this gap, a national group of subject matter experts (SMEs) from diverse fields collaborated to develop an evidence-based methodology and simple user-friendly tool for evaluating burn prevention and fire safety messages.

**Methods:**

In 2021, eleven SMEs gathered under a FEMA-funded two-year grant project. Extensive reviews were conducted of medical, prevention, behavioral science, psychology, public health, and education literature to compile key findings and approaches. The identified components of evidence-informed effective messaging were utilized to create an online messaging reference guide and a 29-question self-evaluation tool (IMPAC-Tool). The online questionnaire-based format of IMPAC-Tool auto-scored the message being assessed and provided specific recommendations for improvement. The reference guide included the supporting evidence, examples, additional resources, and tips to assist users. Pilot testing was conducted via a national stakeholder focus group instructed to use IMPAC-Tool to evaluate safety messages currently embedded in their prevention programs. Testing results and feedback were utilized to revise and improve the tool.

**Results:**

In the pilot test of key stakeholders (n=15), 90% of respondents stated the tool questions were helpful in evaluating and improving their safety messages. Based on feedback, minor revisions were made to the tool for clarity. A beta test was conducted via a large national community risk reduction network (n=56 organizations), with 13 providing focus group responses and 29 completing the tool evaluation. Half of the messages evaluated in IMPAC-Tool scored < 10% (< 2 points out of 17) in quality. Qualitative results showed IMPAC-Tool prompted them to rethink their message, revise it to be action-focused, change from “don’t” to “do” messaging, and incorporate special needs, cultural, and accessibility considerations. The free online IMPAC-Tool was publicly launched in Fall 2023, with an additional 45 responses from injury, trauma, and burn prevention fields.

**Conclusions:**

All injury and violence prevention programs can employ the evidence-based IMPAC-Tool to evaluate the efficacy, potential effectiveness, and specific areas for enhancement of their safety messages.

**Applicability of Research to Practice:**

This project provides a foundation for future studies to evaluate the effectiveness of evidence-based messaging in directly preventing injuries and deaths.

**Funding for the Study:**

Federal Emergency Management Agency (FEMA) Fire Prevention and Safety Grant EMW -2020-FP00029

